# Comprehensive exploration of the expression and prognostic value of AQPs in clear cell renal cell carcinoma

**DOI:** 10.1097/MD.0000000000029344

**Published:** 2022-10-14

**Authors:** Huanrui Wang, Weiyu Zhang, Zehua Ding, Tao Xu, Xiaopeng Zhang, Kexin Xu

**Affiliations:** a Department of Urology, Peking University People's Hospital, Beijing, China; b Peking University Applied Lithotripsy Institute, Peking University, Beijing, China; c Urology and Lithotripsy Center, Peking University People's Hospital, Beijing, China.

**Keywords:** AQPs, ccRCC, expression, immunomodulatory, prognosis

## Abstract

Aquaporins (AQPs) are a family of membrane water channels that facilitate the passive transport of water across the plasma membrane of cells in response to osmotic gradients created by the active transport of solutes. Water-selective AQPs are involved in tumor angiogenesis, invasion, metastasis and growth. However, the polytype expression patterns and prognostic values of eleven AQPs in clear cell Renal Cell Cancer (ccRCC) have yet to be filled.

We preliminarily investigated the transcriptional expression, survival data and immune infiltration of AQPs in patients with renal cell cancer via the Oncomine database, Kaplan–Meier Plotter, UALCAN cancer database, and cBioPortal databases.

The ethical approval was waived by the local ethics committee of Peking University People's Hospital for the natural feature of mine into databases.

The mRNA expression of AQP1/2/3/4/5/6/7/11 was significantly decreased in ccRCC patients. Meanwhile, MIP and AQP1/2/4/6/7/8/9/11 are notably related to the clinical stage or pathological grade of ccRCC. Lower levels of AQP1/3/4/5/7/10 expression were related to worse overall survival (OS) in patients diagnosed with ccRCC. The AQP mutation rate was 25% in ccRCC patients, but genetic alterations in AQPs were unlikely to be associated with OS and disease free survival in ccRCC patients. In addition, the expression of AQP1, AQP3, AQP4 and AQP10 was positively correlated with immune cells, and the expression of AQP6, AQP7 and AQP11 was negatively correlated with immune cells. AQP9 had a strong and significantly positive correlation with multiple immune cells.

Abnormal expression of AQPs in ccRCC indicated the prognosis and immunomodulatory state of ccRCC. Further study needs to be performed to explore AQPs as new biomarkers for ccRCC.

## 1. Introduction

Aquaporins (AQPs) are composed of a group of genes encoding a family of membrane water channels responsible for the transport of water and some small solutes, such as glycerol, even gas and ions. They are involved in tumor angiogenesis and spread. The AQPs, in general, are subdivided into 3 groups according to their permeability profile and sequence homology.^[1]^ The classical water-selective channels include MIP, AQP1, AQP2, AQP4, AQP5, AQP6, and AQP8. Aquaglyceroprins, including AQP3, AQP7, AQP9, and AQP10, are permeable to water and some small uncharged solutes. A third subfamily, superaquaporins, including AQP11 and AQP12, has low homology at their amino acid level with other AQPs and retains functions and regulation to be fully defined. These subfamilies overlapped functionally. Because of the extremely low expression of AQP12 in renal tissues, it was excluded from the present study.

AQPs are widely expressed in the body, particularly in cell types that are involved in fluid transport, such as epithelial cells in several organs, as well as in some cell types that do not have an obvious role in fluid transport, such as adipocytes.^[2]^ The expression of AQPs manifests in disorder among diverse human malignancies, including breast,^[3,4]^ lung, colorectal,^[4]^ brain,^[4]^ liver^[5]^ and pancreatic^[6]^ cancers, mostly playing a role in tumor types, grades, tumor-associated edema, tumor cell migration and proliferation, cell–cell adhesiveness, and tumor angiogenesis in hematological and solid tumors.^[4,7]^ The functions of AQPs in clear cell RCC (ccRCC) are rarely reported.^[8]^

Renal cell carcinoma (RCC) denotes cancer originating from the renal epithelium and accounts for >90% of cancers in the kidney.^[9,10]^ Targeted therapies^[11]^ (agents targeting the VEGF/PDGFR/mTOR pathway, for instance), immunotherapy^[12]^ (interferon α, high-dose interleukin-2, antibodies against programmed cell death protein 1 ligand 1, PDL1, and antibodies against programmed cell death protein 1, PD1, for instance), and combinations were used for the treatment of metastatic RCC. ccRCC is the most common subtype of renal cancer, accounting for the majority of deaths from kidney cancer. A large genomic analysis has been undertaken and has led to the identification of several novel prevalent mutations in ccRCC, including polybromo 1 (PBRM1, 29%–41% of tumor samples), histone-lysine N-methyltransferase SETD2 (SETD2, 8%–12%), BRCA1-associated protein 1 (BAP1, 6%–10%), lysine-specific demethylase 5C (KDM5C, 4%–7%) and mammalian target of rapamycin (mTOR, 5%–6%).^[13]^ Increasing interest was focused on immune infiltration since immunotherapy improved the outcome of ccRCC,^[14]^ which had the highest T cell infiltration score among 19 cancers.^[15]^ With a deep view of T cell immunosuppression phenotypes, there were links between a tumor-associated macrophage (TAM) phenotype and populations of regulatory T cells and CD8^+^ immunosuppressed T cells, and the composition of immune cells was correlated with progression-free survival.^[16]^

Since the discovery of AQP1, 13 AQPs (MIP, AQP1-12), which are distributed widely in specific cell types in various organs and tissues, have been characterized in humans. AQPs are thought to play distinct and complex roles in multiple human cancers. However, the function of disparate AQPs in the initiation and progression of ccRCC is still ambiguous, and the prognostic value of AQPs in ccRCC remains to be comprehensively investigated. On the basis of the wide application of microarray technology, the expression level and mutations of individual AQPs in patients diagnosed with ccRCC were analyzed in detail to explore the expression profile, potential biological functions, and definite prognostic values of AQPs in ccRCC.

## 2. Methods

### 2.1. Expression analysis using data from ONCOMINE datasets

Data provided by public datasets on ONCOMINE (http://www.oncomine.org) were used for mRNA expression level analyses.^[17]^ Oncomine consists of previously published microarray data and is publicly available. The mRNA expression levels of MIP and AQP1/2/3/4/5/6/7/8/9/10/11 were analyzed. In the present study, the data type was set as mRNA, the top 10% gene rank was included, the *P* value was set to .01, and the fold change was set as 1.5. The expression of genes in the 2 panels, cancer tissues and corresponding normal samples, was compared.

### 2.2. Clinicopathological analysis using UALCAN from TCGA dataset

The UALCAN platform (http://ualcan.path.uab.edu) is a comprehensive and interactive web resource that provides authoritative and reliable gene expression and clinicopathological data of 31 types of cancer.^[18]^ In the present study, the mRNA expression of 12 AQPs in ccRCC tissues and their relationship with clinicopathological parameters (cancer stage and grade) were analyzed using the UALCAN database with 72 normal cases and 533 tumor cases. For clinical analysis, the study included 72 normal cases, 267 cases at stage 1, 57 at stage 2, 123 at stage 3 and 84 at stage 4. For pathological analysis, the study included 72 normal cases, 14 cases at grade 1, 229 at grade 2, 206 at grade 3 and 76 at grade 4. Cases with any data loss or corruption of cancer stage or grade were excluded from the corresponding analyses.

### 2.3. Survival analysis using the Kaplan–Meier (KM) Plotter

The KM Plotter (http://www.kmplot.com) is an online database capable of assessing the effect of 54k genes on survival in 21 cancer types.^[19]^ Overall survival (OS) acts as the gold standard primary endpoint to evaluate the outcome of cancers, and relapse-free survival (RFS) refers to the length of time after primary treatment for a cancer ends that the patient survives without any signs or symptoms of that cancer. In the present study, the expression of AQPs was divided into 2 cohorts according to the Auto Select best cutoff. Based on that, OS and RFS were used to appraise the prognostic values of MIP and AQP1-11 in ccRCC. The number of cases included in the analysis is marked below each figure.

### 2.4. Genetic mutations analysis using the cBioPortal

The cBioPortal database (http://www.cbioportal.org) hosts cancer genomics studies and provides an open resource for exploring, visualizing and analyzing cancer research.^[20]^ cBioPortal was used to explore genetic mutations and their correlation with the OS and PFS of ccRCC patients.

### 2.5. Immune infiltration analysis

The infiltration levels of immune cell types were quantified by ssGSEA method using gsva package (v1.34.0) in R software. The following 24 types of immune cells were obtained: activated DCs (aDCs), B cells, CD8 T cells, cytotoxic cells, DCs, eosinophils, immature DCs (iDCs), mast cells, neutrophils, macrophages, NK cells, NK CD56bright cells, NK CD56dim cells, plasmacytoid DCs (pDCs), T cells, T helper cells, T central memory (Tcm), T effector memory (Tem), T gamma delta (Tgd), T follicular helper (Tfh), Th1 cells, Th2 cells, Th17 cells, and Tregs.

### 2.6. Validation at the protein level by using the human protein atlas (HPA) database

The databases used above explored the expression of AQPs at the mRNA level, so we retrieved the data from the HPA database to assess the expression of AQPs at the protein level. The HPA database contains immunohistochemistry images of a wide variety of cancers, providing protein expression profiles. The HPA database analyzed the proteome of 17 major cancers by using clinical metadata and genome-wide transcriptomics data of nearly 8000 patients. Based on the rate of positively stained cells and the staining intensity, the results were scored as strong, moderate, weak and negative.

### 2.7. Statistical analysis

Statistical analyses were performed by ONCOMINE, UALCAN, KM Plotter and cBioPortal. KM method and log-rank test were adopted for survival analysis by calculating hazard ratio (HR) and 95% confidence interval (CI). Quantitative variables were compared using Student *t* test. For the correlation analysis, the Pearson correlation coefficient analysis was used. Two-sided *P* value threshold was set as 0.05 to be statistically significant.

## 3. Results

### 3.1. Disordered transcriptional expression of AQPs in patients with RCC

**Figure 1.**
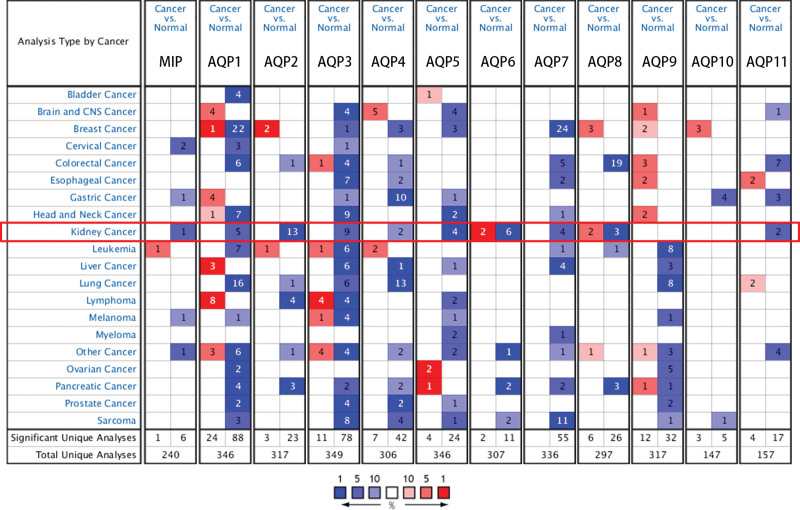
The transcriptional expression of AQPs in various types of cancers (Oncomine). AQP expression at the mRNA level was analyzed by using data from the ONCOMINE database (cancer vs corresponding normal kidney tissue). Significant overexpression is marked in red, and reduced expression is marked in blue. The number in each cell represents the number of analyses that meet the threshold.

Twelve AQPs have been identified in kidney tissues. The transcriptional expression of AQPs (kidney cancer vs. normal kidney tissue) was evaluated using the ONCOMINE database (Fig. 1). Significant overexpression is marked with red, and reduced expression is marked with blue bn. The number of analyses that met the threshold was seen in each cell. As presented in Supplement Digital Content Table S1, http://links.lww.com/MD2/B24 the mRNA expression of AQPs was lower in renal cancers in 7 datasets to a large extent.^[21–27]^ The mRNA expression level of MIP was significantly downregulated in patients with papillary renal cell carcinoma in one dataset (fold change = −2.368).^[21]^ AQP1 was expressed at lower levels in chromophobe renal cell carcinoma (fold change = −11.166), renal pelvis urothelial carcinoma (fold change = −7.221), renal Wilms tumor (fold change = −13.299), chromophobe renal cell carcinoma (fold change = −14.864), and nonhereditary clear cell renal cell carcinoma (fold change = −4.503) samples than in corresponding normal samples. AQP2 was expressed at lower levels in ccRCC (fold change = −4.592, Jones Renal, −13.183, Yusenko Renal, −5.590, Gumz Renal, -2.567, Lenburg Renal), renal Wilms tumor (fold change = −26.722), chromophobe renal cell carcinoma (fold change = −4.758, Jones Renal, −13.013, Yusenko Renal), renal oncocytoma (fold change = −21.537), papillary renal cell carcinoma (fold change = −4.945, Jones Renal, −14.794, Yusenko Renal), nonhereditary clear cell renal cell carcinoma (fold change = −11.212), and hereditary clear cell renal cell carcinoma (fold change = −12.336) versus corresponding normal samples. AQP3 was expressed at lower levels in clear cell renal cell carcinoma (fold change = −3.889), chromophobe renal cell carcinoma (fold change = −1.887, Jones Renal, −5.181, Higgins Renal), papillary renal cell carcinoma (fold change = −8.716, Jones Renal, −9.131, Higgins Renal), nonhereditary clear cell renal cell carcinoma (fold change = −3.699), hereditary clear cell renal cell carcinoma (fold change = −2.391), renal oncocytoma (fold change = −13.367), and clear cell sarcoma of the kidney (fold change = −8.523) versus corresponding normal samples. AQP4 was expressed at lower levels in clear cell renal cell carcinoma (fold change = −1.961) and renal oncocytoma (fold change = −6.349) samples than in corresponding normal samples. AQP5 was expressed at lower levels in granular renal cell carcinoma (fold change = −1.803), chromophobe renal cell carcinoma (fold change = −2.080), papillary renal cell carcinoma (fold change = −1.508), and renal oncocytoma (fold change = −1.759) samples than in normal samples. AQP6 was found to be expressed at lower levels in clear cell renal cell carcinoma (fold change = −8.330, Yusenko renal, −4.140, Gumz renal), papillary renal cell carcinoma (fold change = −2.013, Jones renal, −5.431, Yusenko renal), renal Wilms tumor (fold change = −3.621), and renal pelvis urothelial carcinoma (fold change = −1.750) versus corresponding normal samples. AQP7 was expressed at lower levels in clear cell renal cell carcinoma (fold change = −9.610, Gumz Renal, −2.102, Lenburg Renal), renal oncocytoma (fold change = −2.692), and renal pelvis urothelial carcinoma (fold change = −1.741) samples than in normal samples. AQP8 was found to be expressed at lower levels in clear cell renal cell carcinoma (fold change = −3.097), renal pelvis urothelial carcinoma (fold change = −2.198), and papillary renal cell carcinoma (fold change = −2.042) samples than in corresponding normal samples. AQP11 was expressed at lower levels in clear cell renal cell carcinoma (fold change = −1.944) and chromophobe renal cell carcinoma (fold change = −4.694) samples than in corresponding normal samples.

### 3.2. Relationship between the mRNA expression of AQPs and the clinicopathological furfures of patients with ccRCC

**Figure 2.**
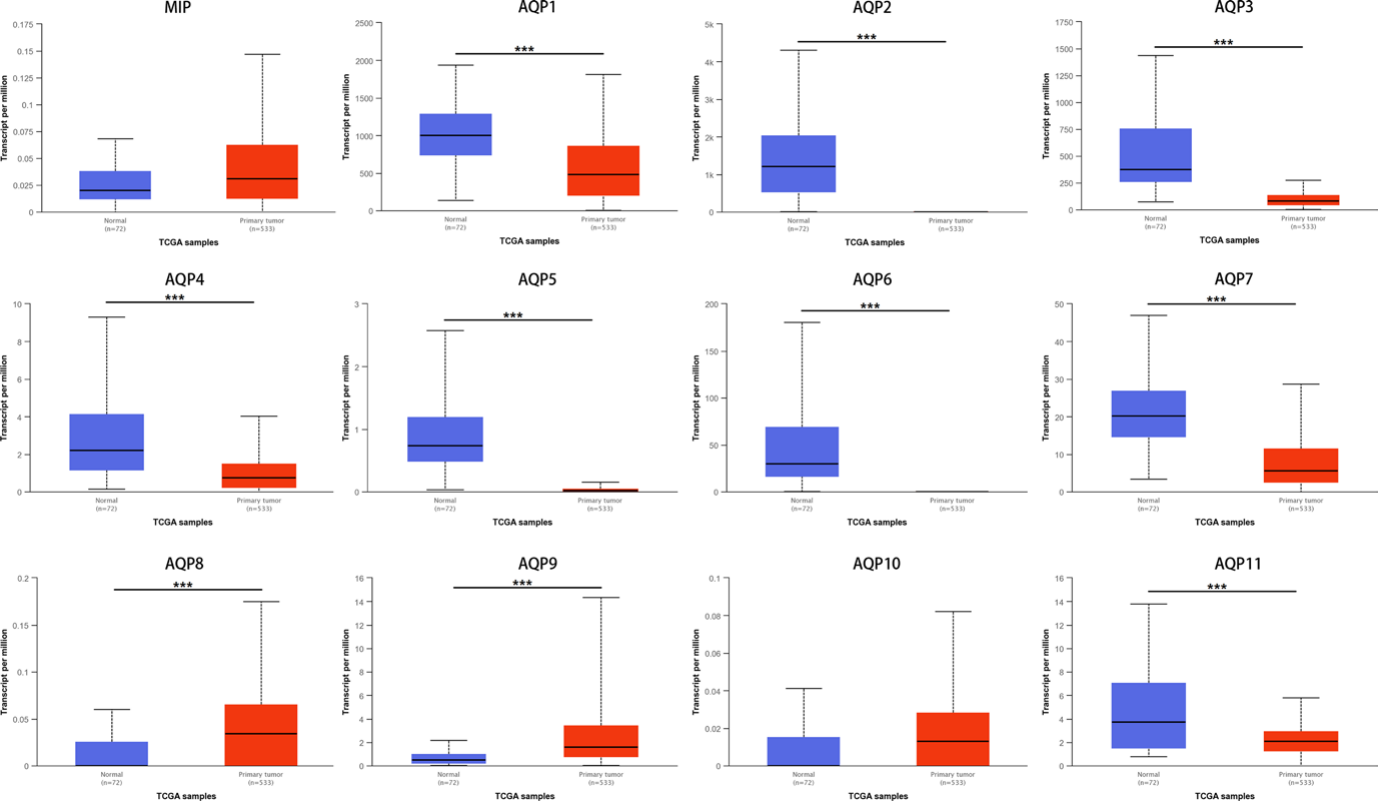
The abnormal mRNA expression of AQPs in cancer and corresponding normal kidney tissues results from the TCGA cohort (UALCAN). The expression of AQP8 and AQP9 in cancer tissues increased compared with corresponding normal tissues, while AQP1-7 and AQP10 decreased. and MIP and AQP10 remained not significantly different. ***P < .001.

**Figure 3.**
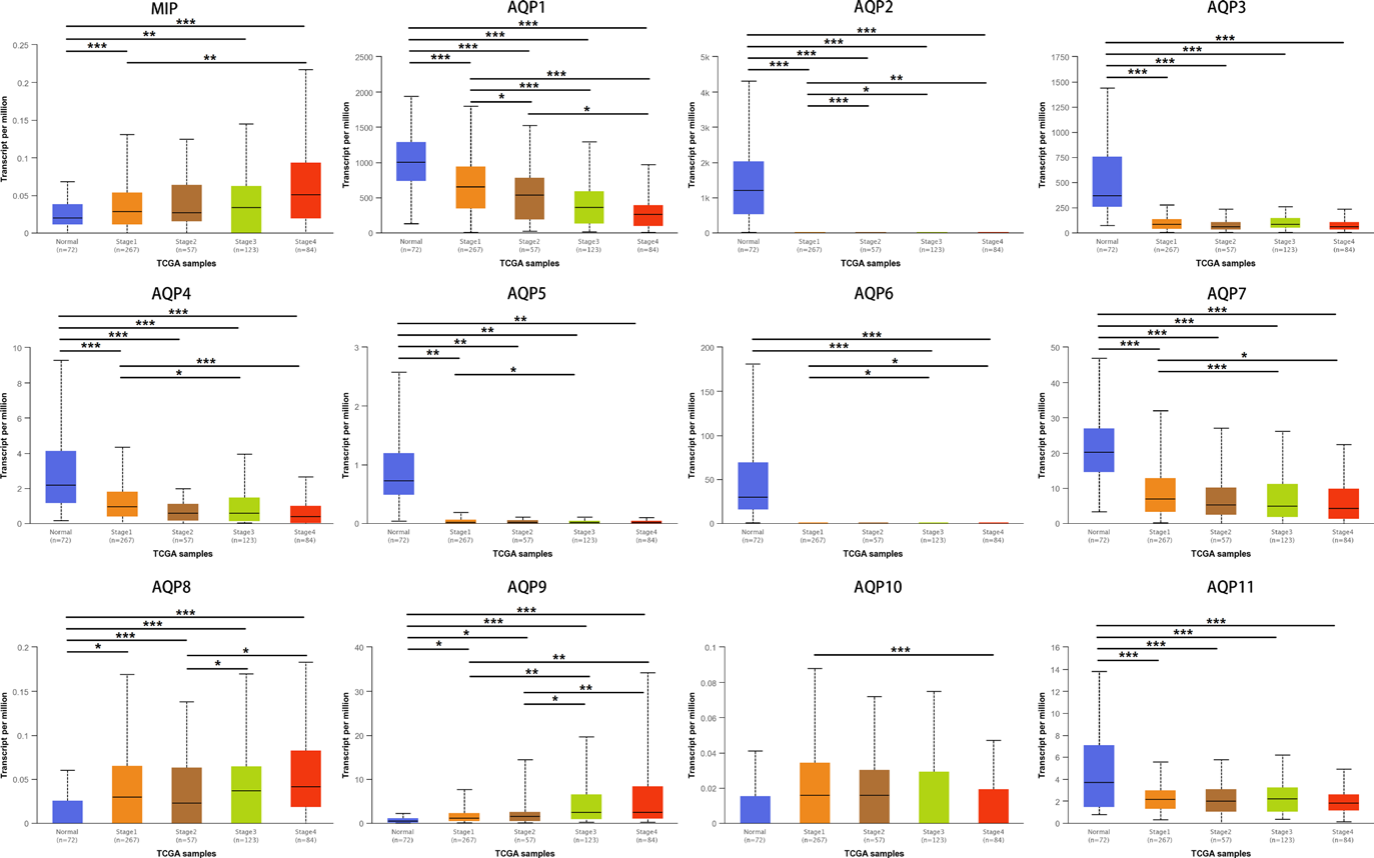
Correlation analysis of AQP mRNA expression and cancer stages was conducted by using UALCAN. The results showed that the mRNA expression level of AQPs was significantly associated with the clinical features of patients diagnosed with ccRCC. *P < .05, **P < .01, ***P < .001.

**Figure 4.**
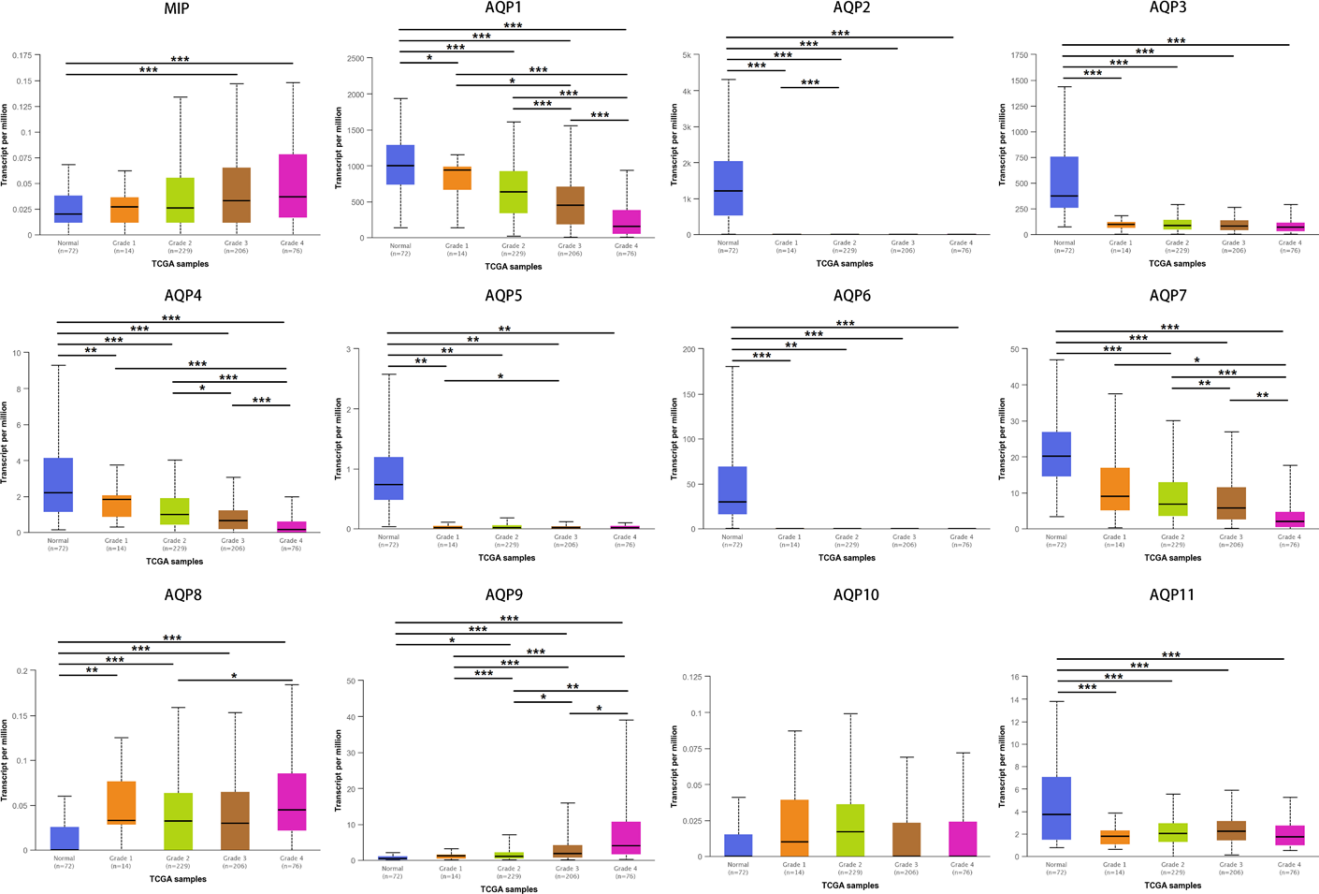
AQP expression varied with pathological grade. The expression of AQP8 and AQP9 increased as pathological grade rose, while AQP1, AQP4, and AQP7 showed a negative correlation with pathological grade. *P < .05, **P < .01, ***P < .001.

Using the UALCAN platform, the mRNA expression of AQPs between ccRCC and renal tissues was compared. In the present study, the results from UALCAN revealed that the mRNA expression of AQP1, AQP2, AQP3, AQP4, AQP5, AQP6, AQP7, and AQP11 was remarkably lower in ccRCC samples than in corresponding normal renal tissues, and the expression of MIP, AQP8, AQP9, and AQP10 was higher in the former than in the latter (Fig. 2). We also analyzed the expression of AQPs with tumor stage and grade for ccRCC. As displayed in Figure 3, the expression of 12 AQPs had a close association with the cancer stages of ccRCC patients. The expression of MIP, AQP8, and AQP9 was significantly higher in the late stage (stages 3–4), and the expression of AQP1, AQP4, AQP7, and AQP10 was significantly lower in the late stage (stages 3–4). The expression of AQP3 and AQP11 remained close in different stages. Similarly, as illustrated in Figure 4, box plots showed that AQP mRNA expression was prominently associated with pathological grade. The mRNA expression of AQP8 and AQP9 tended to be higher, and the pathological grade increased. Conversely, the mRNA expression of AQP1, AQP4 and AQP7 was negatively correlated with pathological grade in ccRCC. Overall, the UALCAN results indicated that the mRNA expression of AQP1, AQP4, AQP7 and AQP9 in ccRCC patients was significantly correlated with clinicopathological furfures.

### 3.3. Verification at protein level of AQPs

**Figure 5.**
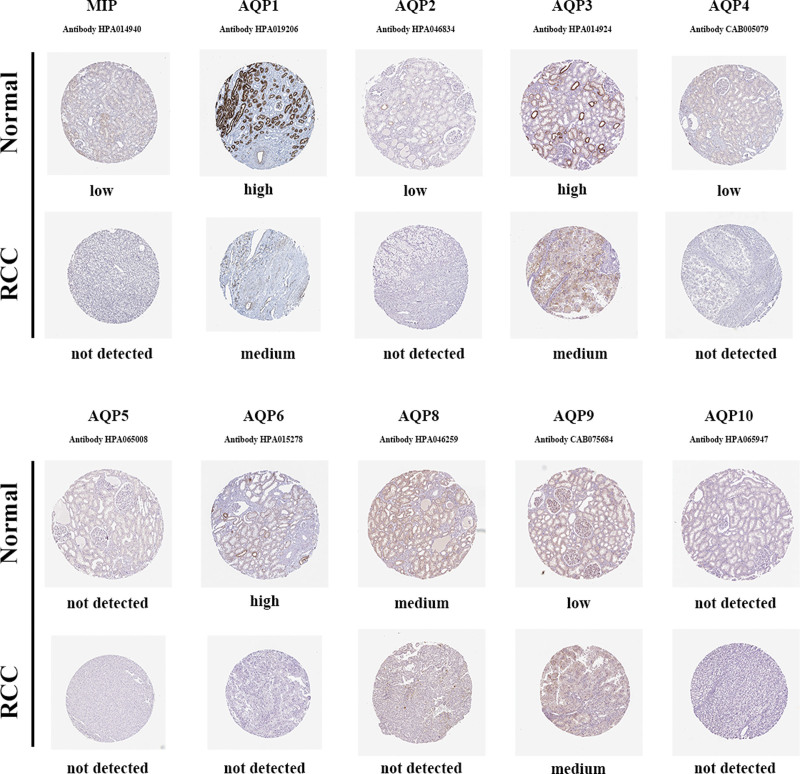
Immunohistochemical images from the HPA database showed AQP expression at the protein level in ccRCC and corresponding normal tissues.

Immunohistochemically stained images from the HPA database showed AQP protein expression in ccRCC tissues and corresponding normal tissues (Fig. 5). The expression was marked as high, medium, low and not detected in the figure. These results for AQP1-4, 6, and 9-10 were consistent with the transcriptional analysis.

### 3.4. Prognostic values of AQPs in ccRCC

**Figure 6.**
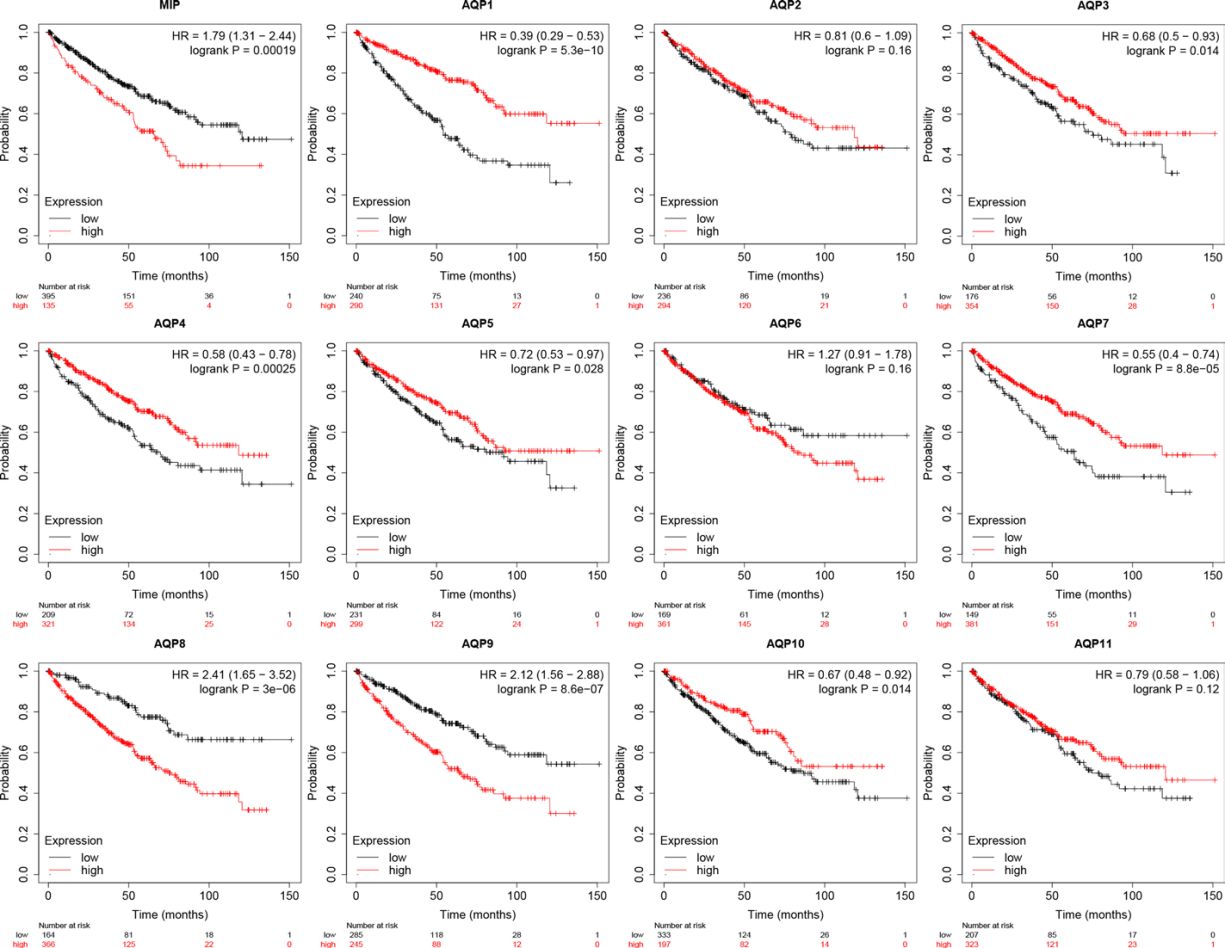
The OS and RFS of patients with ccRCC are presented with survival curves via Kaplan–Meier Plotter. Among the twelve AQP family members, low mRNA expression of AQP1, AQP3, AQP4, AQP5, AQP7, AQP10, and AQP11 was associated with worse OS in ccRCC patients. Low MIP, AQP8, and AQP9 were correlated with longer OS time. AQP2 and AQP6 showed no significant difference.

**Figure 7.**
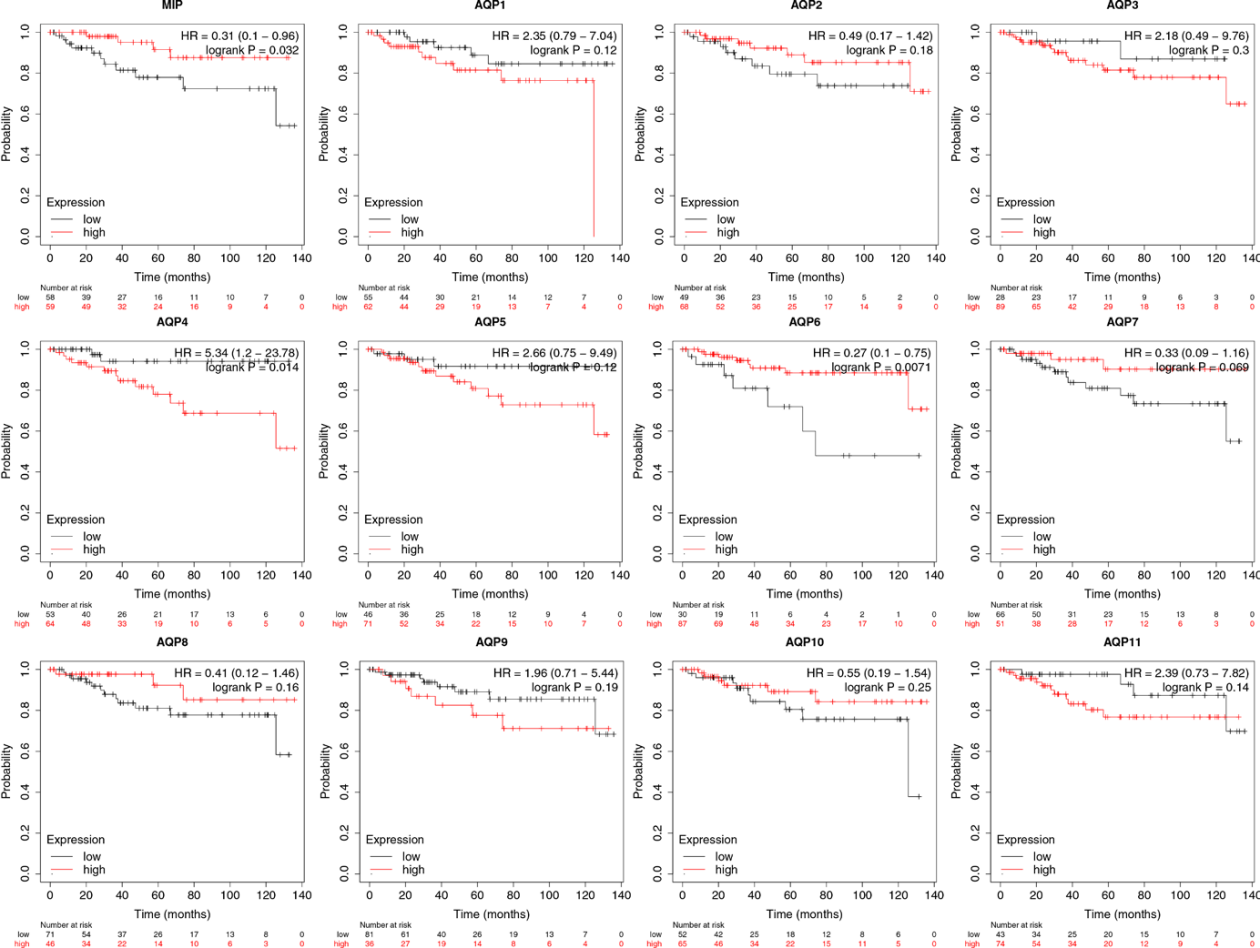
Relationships between mRNA expression levels of twelve AQP family members and the relapse-free survival (RFS) of patients with ccRCC. Analyses were conducted using Kaplan–Meier plotter.

Survival curves were used to show the overall survival (OS) times of ccRCC patients by using Kaplan–Meier Plotter (Fig. 6). Among the twelve AQP family members, low mRNA expression of AQP1 (*P* = 5.3e-10), AQP3 (*P* = .014), AQP4 (*P* = .00025), AQP5 (*P* = .028), AQP7 (*P* = 8.8e-05) and AQP10 (*P* = .014) was associated with worse OS in ccRCC patients. Low MIP (*P* = .00019), AQP8 (*P* = 3e-06), and AQP9 (*P* = 8.6e-07) showed a relationship with longer OS time. AQP2 and AQP6 showed no significant difference. Furthermore, RFS was also analyzed (Fig. 7). The increased mRNA expression of MIP (*P* = 0.032) and AQP6 (*P* = .0071) and the decreased mRNA expression of AQP4 (*P* = .014) were significantly associated with longer RFS.

### 3.5. AQP genetic alteration analyses in ccRCC patients via the cBioPortal database

**Figure 8.**
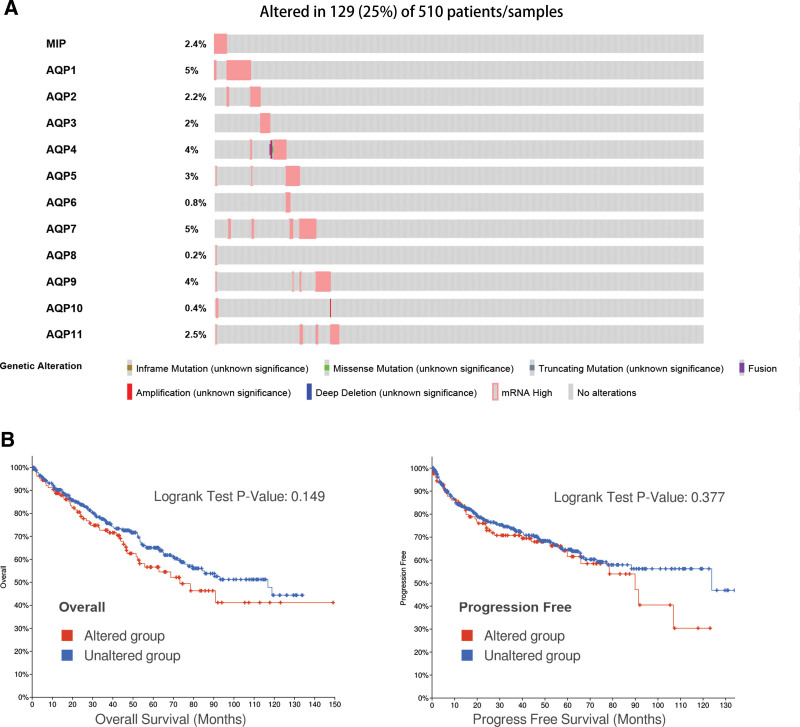
Relationships between AQP gene mutations and survival in ccRCC patients. (A) The AQP mutation rate was 25% in ccRCC patients. The top 5 highest mutation rates were present in AQP1 (5%), AQP7 (5%), AQP9 (4%), AQP4 (4%), and AQP5 (3%). (B) Genetic alterations in AQPs were unlikely to be associated with overall survival time (OS, left) and disease-free survival (DFS, right) in ccRCC patients.

Genetic alterations in AQPs were analyzed by using cBioPortal databases. The correlations with OS and PFS of ccRCC patients were explored. In the present study, 510 ccRCC patients were enrolled, and their AQP gene mutation information was thoroughly analyzed. The AQPs mutation rate was 31.5% in ccRCC patients. As shown in Figure 8A, the percentages of genetic alterations in AQPs of ccRCC ranged from 0.2 to 5% for single genes (MIP, 2.4%, AQP1, 5%, AQP2, 2.2%, AQP3, 2%, AQP4, 4%, AQP5, 3%, AQP6, 0.8%, AQP7, 5%, AQP8, 0.2%, AQP9, 4%, AQP10, 0.4%, AQP11, 2.5%). The results of KM curves and log-rank tests suggested that genetic alterations in AQPs were correlated with worse OS (*P* = .149) and PFS (*P* = .377) of patients with ccRCC (Fig. 8B). In short, genetic alterations in AQPs slightly influenced the prognosis of ccRCC patients.

### 3.6. Tumor-infiltrating immune cells associated with AQPs

**Figure 9.**
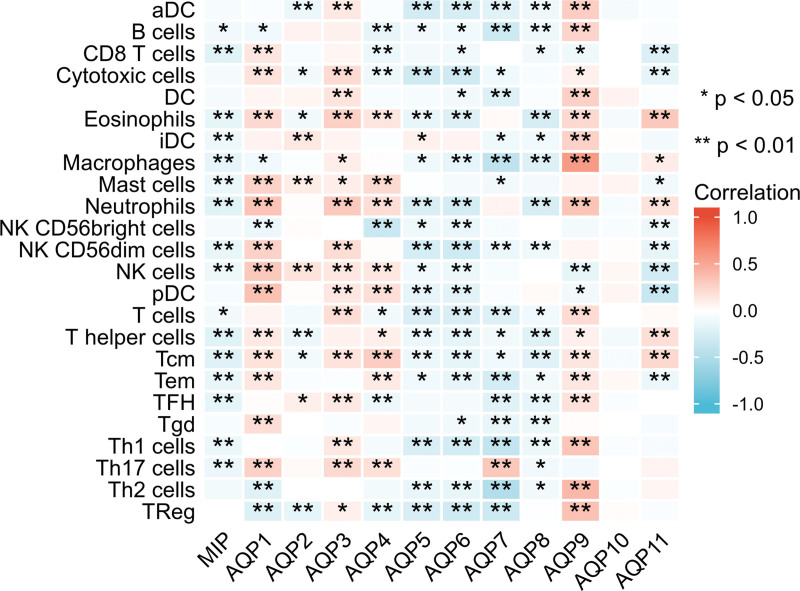
Tumor-Infiltrating Immune Cells Associated with AQPs. AQP family genes were associated with immune-cell subset. Red boxes indicate positive correlation and blue boxes indicate negative correlation. *, *P* < .05; **, *P* < .01. aDC = activated DC, iDC = immature DC, pDC = plasmacytoid DC, Tcm = T central memory, Tem = T effector memory, Tfh = T follicular helper, Tgd = T gamma delta.

The expression of AQP9 had a strong and significantly positive correlation with multiple immune cells. The expression of AQP1, AQP3, AQP4, and AQP10 was positively correlated with immune cells. The expression of AQP6, AQP7, and AQP11 was negatively correlated with immune cells. The potential immunological correlation between AQPs and tumor-infiltrating immune cells was studied. The expression levels of MIP, AQP1, AQP2, AQP3, AQP4, AQP5, AQP6, and AQP9 were negatively correlated with tumor purity (*P* < .05), suggesting that these AQPs were highly expressed in the ccRCC microenvironment (Fig. 9).

## 4. Discussion

AQPs are a family of proteins that act as water and solute channels and play essential roles in cell proliferation, migration, differentiation and apoptosis. Increasing attention and focus have been drawn to the relevance of AQPs and cancer progression.^[28]^ Recent evidence has indicated that several AQPs are involved in the biological processes of tumor growth, angiogenesis and metastatic processes,^[29]^ and AQP overexpression was previously reported in over 12 different tumor cell types.^[30]^ Since ccRCC was knew well as a highly immune-infiltrated tumor, results from the present study showed that AQP family genes were associated with immune-cell subset, implying an effect on immunoflofosis in tumor microenvironment and acted as candidate prognostic factor for patients with ccRCC, which was similar to some of literatures report.^[31]^ The present study has, for the first time, comprehensively analyzed the expression of AQPs in ccRCC, explored the prognostic values of different AQPs in ccRCC and might contribute to the treatment strategy.

The main distribution of MIP was in the fiber cells of the eye lens, which worked to maintain homeostasis and transparency of the lens. A high level of MIP mRNA was associated with poor OS in all histological subtype gastric cancer patients, HR = 1.55 (1.29–1.86)^[32]^ and all ovarian cancer patients, HR = 1.15 (1.01–1.31),^[33]^ but better RFS in all histological subtype breast cancer.^[34]^ The expression of MIP is extremely low in the kidney, and the biological performance of MIP in ccRCC has not yet been studied. In the results from the present study, the transcriptional level of MIP in ccRCC tissues was the lowest among all AQPs. Datasets from Oncomine and TCGA revealed that the expression level of MIP in ccRCC was higher than that in corresponding normal renal tissues. The expression of MIP was associated with the clinical stage and pathological grade. Moreover, after follow-up for 150 months, higher expression of MIP was significantly associated with poor OS and RFS.

AQP1, a classical water channel widely distributed in the body, plays a central role in the regulation of water transport. AQP1 was revealed to be involved in the regulation of angiogenesis, cell migration and cell growth.^[29]^ AQP1 occupied the largest proportion of studies on AQP function in a diverse array of cancer types.^[35]^ Elevated expression levels of AQP1 in urine were detected in renal cell carcinoma patients,^[36,37]^ and urinary concentrations of AQP1 in renal cell carcinoma correlated with tumor size and stage.^[38]^ According to the present study, the mRNA expression of AQP1 was significantly downregulated in ccRCC tissues from 5 datasets. A box plot was used to show a comparatively low level of AQP1 in ccRCC tissues compared with normal tissues. As clinical stages and pathology grade advanced, the expression of AQP1 decreased with statistical significance. Via multivariable survival analysis, AQP1 emerged as the best candidate among AQPs to be a prognostic and survival biomarker for ccRCC.

AQP2, the vasopressin-regulated water channel involved in urine concentration, determines the water permeability of the kidney collecting duct. AQP2 was E2 dose-dependently increased and expressed in endometrial carcinoma tissues, which was blocked by an estrogen receptor inhibitor, and AQP2 knockdown attenuated E2-enhanced migration, invasion, and adhesion of IK cells.^[39]^ Quantitative RT–PCR revealed that AQP2 was decreased 5.18 times in 127 RCC patients (*P* < .05), and with the promotion of staging, the expression of AQP2 was reduced gradually (*P* < .05).^[40]^ The same expression trend was observed in our present study and 13 previous studies. However, the OS and RFS of patients with high or low AQP2 were not significantly different. Based on the regulation of the biological metabolism of water, AQP2 was reasonably assumed to participate in the tumor microenvironment. Further study deserved to be performed.

AQP3 has a wide tissue distribution. In renal tissue, AQP3 is localized in the basolateral plasma membranes of cortical and outer medullary collecting duct principal cells.^[41]^ AQP3 was recently shown to be stimulated by EGF signaling and transport H_2_O_2_ through the plasma membrane, contributing to the initiation of intracellular signaling in cell motility, inflammation, metastasis, proliferation and epithelial-to-mesenchymal transition.^[42]^ In addition, AQP3 acted as an oncogenic gene in liver cancer, which promoted the stimulation and nuclear translocation of signal transducer and activator of transcription 3 with subsequent accelerated CD133 transcription.^[43]^ Decreased expression of AQP3 in ccRCC tissues was observed in 9 databases. After analyses synthetically, AQP3 was downregulated in ccRCC with significant differences and decreased with clinical stage and pathological grade. Higher expression of AQP3 was related to longer OS (*P* = .014).

AQP4 is a predominant AQP located in the central nervous system, enables central nervous system fluid homeostasis and promotes maintenance of the blood–brain barrier.^[44]^ In a noncancerous epithelial cell line, AQP4 was found to have a significant effect on collective migration. The decrease in epithelial markers following AQP4 expression was not associated with epithelial-to-mesenchymal transition because lower vimentin levels were also observed upon AQP4 expression, which implies how AQP4 functions in cell–cell adhesion.^[7]^ Upregulation of AQP4 protein and RNA in glioma has been detected, contributing to the invasiveness and migration of glioma cells.^[45]^ AQP4 dysregulation in renal cancer has not yet been reported. In the present study, the expression of AQP4 was downregulated significantly in ccRCC tumor tissues and decreased with increasing clinical stage and pathological grade.

Similar to AQP1 and AQP3, AQP5 plays a role in the activation of cell proliferation and resistance to oxidative stress by facilitating H_2_O_2_ diffusion through cell membranes^[46]^ and preferential polarization in the leading edge of migrating cells.^[47]^ The expression of AQP6 changes with the development of renal cell carcinoma and oncocytoma, but the underlying mechanism remains to be further studied.^[48]^ Four datasets proved the lower expression of AQP5 in ccRCC tumors than in peritumoral tissues. Upregulated expression of AQP6 in ccRCC tumor tissues was observed in 2 datasets, while downregulated expression was observed in 6 datasets. By integrating these data, the expression of AQP5 and AQP6 was found to be significantly lower in tumor tissues but inapparent to clinical stage and pathological grade.

AQP7 is abundantly expressed in adipose tissue, mediating the efflux of newly generated glycerol and contributing to the development of metabolic disease.^[49]^ However, the relationship of AQP7 with renal cancer has not been explored. Lower expression of AQP7 in ccRCC tumor tissues was found in 4 datasets, and recombinational data analyses showed an obvious downregulation of AQP7 expression in ccRCC. As the pathological grade increased, the expression of AQP7 decreased.

Abnormal expression of AQP8 was detected in cervical cancer, leukemia, and esophageal cancer. It was reported that AQP8 acted as an H_2_O_2_ transport facilitator across the plasma membrane of B1647 cells, a model of acute myeloid human leukemia, and was inhibited by sulforaphane.^[50]^ The exact mechanism in renal cancer is not yet clear. Tow datasets showed higher expression of AQP8 in ccRCC and 3 showed lower expression. The total data showed a significant increase in the expression of AQP8 in ccRCC tumor tissues.

AQP9 expressed in acute myeloid leukemia was permeable to As_2_O_3_, and upregulated AQP9 enhanced cytotoxicity in acute myeloid leukemia cell lines, expanding the therapeutic spectrum of As_2_O_3_.^[51]^ The expression of AQP9 was slightly higher in ccRCC than in normal renal tissue, and the trend went with the progression of clinical stage and pathological grade.

Abnormal expression of AQP10 and AQP11 was reported in gastric cancer,^[32]^ but the regulatory mechanism demanded prompt solution. Expression of AQP10 in ccRCC was flat with normal tissue, so in different clinical stages and pathological grades. Downregulated expression of AQP11 in ccRCC was observed in 2 datasets. By integrating these data, the expression of AQP11 was found to be significantly lower in tumor tissues but inapparent to clinical stage and pathological grade.

Considering the natural characteristics of bioinformatics methodology, different databases include diverse panels of patients and sometimes result in mutually contradictory conclusions. For example, the results showed overexpression of AQP10 in cancer cells compared to normal cells; however, survival analysis indicated that overexpression of this gene increases the patient survival rate. The 2 analyses used data from ONCOMINE datasets and KM Plotter. Moreover, some results from the present study were opposite to previous studies for the same reason. Although contradictory, the results reflected the different constitutions but of real panels of patients. Further study is needed to explore potential biological functions and values.

With limited cases used for immunohistochemistry staining, the protein expression results were partly consistent with the analysis at the transcriptional level. Further study at the protein level with more tissue samples is needed.

## 5. Conclusions

Abnormal expression of AQPs in ccRCC indicated the prognosis and immunomodulatory state of ccRCC, indicating that AQPs are potential biomarkers of ccRCC. Further exploration would be valuable.

## Author contributions

Huanrui Wang, the conception design of the study, data analysis and draft the manuscript.

Weiyu Zhang, the figure editing, manuscript preparation.

Xiaopeng Zhang, critical revision and final approval of the manuscript.

Supervision: Kexin Xu, Xiaopeng Zhang.

Writing – original draft: Huanrui Wang, Weiyu Zhang.

Writing – review & editing: Kexin Xu, Xiaopeng Zhang.

## Acknowledgment

Thanks Prof. Kexin Xu for supervision on the present study and provided some important suggestions. Thanks Dr. Zehua Ding for help in language improvement.

## Supplementary Material


