# Supplementary material for: Comprehensive exploration of the expression and prognostic value of AQPs in clear cell renal cell carcinoma
Source: Medicine (Baltimore). 2022 Oct 14;101(41):e29344. doi: 10.1097/MD.0000000000029344 (PMC9575724; doi:10.1097/MD.0000000000029344)
Supplement: Supplementary file 1 [file medi-101-e29344-s001.docx]

supplement table 1. Significant Changes in AQP Expression at the Transcription Level between Different Types of Kidney Cancer and Normal Kidney Tissues (Oncomine Database)

| supplement table1. The Significant Changes of AQP Expression in Transcription Level between Different Types of Kidney Cancer and Normal Kidney Tissues (Oncomine Database) | | | | | |
| --- | --- | --- | --- | --- | --- |
|  | Type of Kidney cancer vs. Normal Kidney Tissue | Fold change | p Value | t Test | Source and/or Reference |
| AQP0(MIP) | Papillary Renal Cell Carcinoma | -2.368 | 8.23E-18 | -16.990 | Jones Renal[1] |
| AQP1 | Chromophobe Renal Cell Carcinoma | -11.166 | 1.09E-10 | -20.090 | Jones Renal[1] |
|  | Renal Pelvis Urothelial Carcinoma | -7.221 | 2.15E-5 | -7.798 | Jones Renal[1] |
|  | Renal Wilms Tumor | -13.299 | 0.005 | -3.784 | Yusenko Renal[2] |
|  | Chromophobe Renal Cell Carcinoma | -14.864 | 0.005 | -3.438 | Yusenko Renal[2] |
|  | Non-Hereditary Clear Cell Renal Cell Carcinoma | -4.503 | 1.70E-5 | -4.751 | Beroukhim Renal[3] |
| AQP2 | **Clear Cell Renal Cell Carcinoma** | -5.590 | 2.93E-11 | -13.694 | Gumz Renal[4] |
|  | **Clear Cell Renal Cell Carcinoma** | -2.567 | 5.55E-6 | -6.779 | Lenburg Renal[5] |
|  | Renal Wilms Tumor | -26.722 | 5.27E-4 | -5.361 | Yusenko Renal[2] |
|  | Chromophobe Renal Cell Carcinoma | -13.013 | 0.002 | -5.570 | Yusenko Renal[2] |
|  | Renal Oncocytoma | -21.537 | 0.004 | -4.023 | Yusenko Renal[2] |
|  | Papillary Renal Cell Carcinoma | -14.794 | 0.001 | -5.661 | Yusenko Renal[2] |
|  | **Clear Cell Renal Cell Carcinoma** | -13.183 | 0.002 | -5.550 | Yusenko Renal[2] |
|  | Chromophobe Renal Cell Carcinoma | -4.758 | 3.13E-10 | -9.389 | Jones Renal[1] |
|  | Renal Pelvis Urothelial Carcinoma | -5.452 | 2.56E-11 | -11.545 | Jones Renal[1] |
|  | Papillary Renal Cell Carcinoma | -4.945 | 4.57E-11 | -10.590 | Jones Renal[1] |
|  | **Clear Cell Renal Cell Carcinoma** | -4.592 | 1.46E-10 | -10.223 | Jones Renal[1] |
|  | Non-Hereditary Clear Cell Renal Cell Carcinoma | -11.212 | 1.87E-8 | -12.468 | Beroukhim Renal[3] |
|  | Hereditary Clear Cell Renal Cell Carcinoma | -12.336 | 4.06E-8 | -13.460 | Beroukhim Renal[3] |
| AQP3 | **Clear Cell Renal Cell Carcinoma** | -3.889 | 4.56E-6 | -7.538 | Higgins Renal[6] |
|  | Chromophobe Renal Cell Carcinoma | -5.181 | 0.010 | -5.267 | Higgins Renal[6] |
|  | Papillary Renal Cell Carcinoma | -9.131 | 0.004 | -5.424 | Higgins Renal[6] |
|  | Non-Hereditary Clear Cell Renal Cell Carcinoma | -3.699 | 8.93E-10 | -8.449 | Beroukhim Renal[3] |
|  | Hereditary Clear Cell Renal Cell Carcinoma | -2.391 | 9.75E-7 | -5.931 | Beroukhim Renal[3] |
|  | Chromophobe Renal Cell Carcinoma | -1.887 | 7.46E-6 | -5.283 | Jones Renal[1] |
|  | Renal Oncocytoma | -13.367 | 0.003 | -4.696 | Jones Renal[1] |
|  | Papillary Renal Cell Carcinoma | -8.716 | 0.005 | -3.928 | Jones Renal[1] |
|  | Clear Cell Sarcoma of the Kidney | -8.523 | 0.006 | -7.511 | Cutculiffe Renal[7] |
| AQP4 | **Clear Cell Renal Cell Carcinoma** | -1.961 | 8.72E-10 | -7.682 | Jones Renal[1] |
|  | Renal Oncocytoma | -6.349 | 0.006 | -3.368 | Yusenko Renal[2] |
| AQP5 | Granular Renal Cell Carcinoma | -1.803 | 0.002 | -5.323 | Higgins Renal[6] |
|  | Chromophobe Renal Cell Carcinoma | -2.080 | 0.007 | -4.614 | Higgins Renal[6] |
|  | Papillary Renal Cell Carcinoma | -1.508 | 0.010 | -3.525 | Higgins Renal[6] |
|  | Renal Oncocytoma | -1.759 | 8.13E-15 | -14.904 | Jones Renal[1] |
| AQP6 | **Clear Cell Renal Cell Carcinoma** | -4.140 | 1.18E-11 | -14.586 | Gumz Renal[4] |
|  | **Clear Cell Renal Cell Carcinoma** | -8.330 | 1.01E-4 | -7.220 | Yusenko Renal[2] |
|  | Papillary Renal Cell Carcinoma | -5.431 | 3.38E-4 | -5.640 | Yusenko Renal[2] |
|  | Renal Wilms Tumor | -3.621 | 0.007 | -3.330 | Yusenko Renal[2] |
|  | Papillary Renal Cell Carcinoma | -2.013 | 5.43E-10 | -8.565 | Jones Renal[1] |
|  | Renal Pelvis Urothelial Carcinoma | -1.750 | 4.25E-7 | -6.329 | Jones Renal[1] |
| AQP7 | **Clear Cell Renal Cell Carcinoma** | -9.610 | 4.45E-7 | -9.447 | Gumz Renal[4] |
|  | Renal Oncocytoma | -2.692 | 2.67E-16 | -16.783 | Jones Renal[1] |
|  | Renal Pelvis Urothelial Carcinoma | -1.741 | 1.58E-5 | -5.873 | Jones Renal[1] |
|  | **Clear Cell Renal Cell Carcinoma** | -2.102 | 3.26E-4 | -4.863 | Lenburg Renal[5] |
| AQP8 | **Clear Cell Renal Cell Carcinoma** | -3.097 | 2.52E-30 | -29.476 | Jones Renal[1] |
|  | Renal Pelvis Urothelial Carcinoma | -2.198 | 1.02E-16 | -22.638 | Jones Renal[1] |
|  | Papillary Renal Cell Carcinoma | -2.042 | 9.11E-17 | -19.223 | Jones Renal[1] |
| AQP9 | NA | NA | NA | NA | NA |
| AQP10 | NA | NA | NA | NA | NA |
| AQP11 | **Clear Cell Renal Cell Carcinoma** | -1.944 | 9.03E-5 | -5.299 | Lenburg Renal[5] |
|  | Chromophobe Renal Cell Carcinoma | -4.694 | 0.001 | -5.430 | Yusenko Renal[2] |
